# Establishment and Validation of an In Vitro Screening Method for Traditional Chinese Medicine-Induced Nephrotoxicity

**DOI:** 10.1155/2018/2461915

**Published:** 2018-06-28

**Authors:** Zhe Ma, Xuexiao Cao, Xiao Guo, Meng Wang, Xiaoliang Ren, Ranran Dong, Rui Shao, Yan Zhu

**Affiliations:** The Institute of Traditional Chinese Medicine, Tianjin University of Traditional Chinese Medicine, Tianjin 300193, China

## Abstract

Renal injury is among the adverse drug reactions (ADRs) caused by herbal medicine products (HMPs). Traditional Chinese medicines (TCMs) have been practiced for over 2000 years in China and East Asia, and herbs are currently used worldwide for the treatment and prevention of chronic and acute disease. Operetta high content analysis (HCA, PerkinElmer, Waltham, MA, USA), which is an in vitro, sensitive, reproducible, multiparametric screening method, was used to evaluate the cytotoxicity of HMPs in cultures of HEK293 human embryo kidney cells. Cytotoxic results were validated by an animal-based subacute toxicity assay. The renal safety of 18 active pharmaceutical agents from 13 TCM herbs with known nephrotoxic potential was evaluated in HEK293 human embryonic kidney cells. A panel of five parameters, cell viability, nuclear area, nuclear roundness, mitochondrial mass, and mitochondrial membrane potential, was utilized to evaluate drug-induced renal mitochondrial and nuclear injury. HCA can be a useful tool for preclinical screening and postclinical evaluation of HMPs. The nephrotoxicity of diosbulbin B and other HMPs was evident at a concentration as low as 0.01 *μ*M.

## 1. Introduction

Traditional Chinese medicines (TCMs) have been widely applied in East Asia over 2000 years. It gradually shows the trend of globalization with approval by the Dietary Supplement Health and Education Act of 1994 in the USA and registration in Europe under the 2004/24/EC Traditional Herbal Medicinal Products Directive. We pay close attention to their therapeutic efficacy, while having few researches on their side effects. Nowadays, a great lack of consciousness about the safety and unreasonable use of TCMs have caused drug-induced injury event of TCMs to increase. In particular, renal damage accounted for a large proportion of adverse drug reactions (ADRs), like acute kidney injury (AKI), acute tubular necrosis, acute/chronic interstitial nephritis, renal papillary necrosis, Fanconi syndrome, nephrolithiasis, and malignancy [1]. Aristolochic acid (AA) was confirmed to be [2, 3] nephrotoxic and carcinogenic, causing extensive renal interstitial fibrosis, tubular atrophy, and thickening of interlobular and afferent arterioles [4] in clinical and animal experiments [5, 6]. Other herbs and bioactive ingredients like Devil's Claw (*Harpagophytum procumbens* DC. Ex Meisn.), licorice (*Glycyrrhiza glabra* L.), cantharidin [7], and aconitine [8] also have been reported to be toxic to kidneys.

However, unlike HMPs of TCM with health administration approval, toxicological data are frequently not available. Drug nephrotoxicity evaluations mainly involved animal toxicity pathology experiment which cost high experiment materials and longer duration [9] and also are not good enough to predict toxical reaction of human body [10]. With the introduction and implementation of the principle of “3R” (Reduction, Replacement, and Refinement), a large number of studies have been carried out on in vitro cell level [11, 12]. Hu J et al. reported the structure-toxicity relationship of 29 cisplatin derivatives with MTT assay in human renal proximal tubule cells (HK-2) and pig kidney epithelial cells (LLC-PK1). However, single-endpoint measures of cell proliferation for drug nephrotoxicity evaluation were not sufficient. An innovative method with higher throughput and effectiveness was needed. Here, high content analysis (HCA), which was an in vitro cell-based multiparameter assay, had high sensitivity and specificity for identifying the mechanisms involved in drug-induced toxicity [13, 14] and was appropriate for safety evaluation of HMPs. It had already made great progress in the detection of hepatotoxicity. Saito J et al. established an HCA assay in human hepatocyte cell lines (HepaRG and HepG2) and helped to clarify the contribution of metabolism to hepatocyte toxicity.

Mitochondria are the “power house” in a cell, which generate the energy compound ATP via oxidative phosphorylation, so mitochondria have an irreplaceable role in the metabolism of cells. However, there was a general lack of attention to mitochondrial activity in previous renal toxicity studies. CDDP is a potent chemotherapeutic antineoplastic drug that can induce cell injury and death in renal tubules, cause vascular dysfunction, and activate a strong inflammatory response [15–19]. Mitochondrial dysregulation played an important pathogenic role in CDDP nephrotoxicity, especially in renal tubular cell injury and death [20]. CsA is a potent immunosuppressive drug with liver, kidney, endocrine pancreas, and nervous system toxicity. Clinical data and experimental studies had revealed that CsA nephrotoxicity was induced by drug-related oxidative stress [21–26].

The study evaluated 18 ingredients coming from 13 different TCM herbs that were all adopted in marketed HMPs with high sale volume, as shown in Table 1. In the 2015 edition of Chinese Pharmacopoeia, 9 of the 13 herbs, according to their toxicity, were divided into three categories: strong toxicity, toxical, and mild toxicity, without any description of toxicity in kidney, as shown in Table 2. Rhei radix et rhizoma (Dahuang) was one of the four most frequently used herbs in more than 800 Chinese compound prescriptions and their HMPs [27] and was one of the dozen traditional herbs with “global influence” by American scholar. Emodin, the major bioactive ingredient of Dahuang [28], which significantly inhibited the proliferation of HK-2 cells [29], swelled and denatured renal tubule epithelial cell [30]. Moreover, it has been reported that long-time application of total rhubarb anthraquinones (the active therapeutic components in Dahuang) in high dose could cause nephrotoxicity recently. Six-Ingredient Rehmannia Pill (SIRP, also called Liu-wei-di-huang-wan Pill) was one of the most famous restoratives in China with the application history of more than 1000 years. Rhizoma Alismatis (Zexie) was one of six herbs to constitute SIRP, where prolonged use of high doses of Zexie could also lead to nephrotoxicity [31]. The toxical herbs of* Tripterygium wilfordii* radix et rhizoma (Leigongteng) were the most significant therapeutic agent in clinic to treat rheumatism [32].* Mylabris* (Banmao) and its main bioactive ingredient, cantharidin, which belongs to strong toxicity, were reported to cause irritations to skin, mucous membranes, and toxic nephritis with antitumor effect [33–35].

Human embryonic kidney HEK293 cells [36] are specific cell line originating from HEK cells grown in tissue cultures which had been widely used in cell biology as well as toxicology studies [37, 38]. The present study described the establishment of a multiparametric assay of drug renal toxicity in cultured HEK293 and used a novel HCA system that was predictive of drug-induced kidney injury. Three cytotoxic drugs (AA, CsA, and CDDP) were chosen as positive controls for different injury mechanisms, and 18 ingredients that were used widely in HMPs and food were evaluated. Five parameters were taken into account, including cell viability (CV), nuclear area (NA), nuclear roundness (NR), mitochondrial mass (MS), and mitochondrial membrane potential (MMP) as markers of drug-induced renal mitochondrial and nuclear injury.

## 2. Materials and Methods

### 2.1. Reagents and Drugs

Minimum essential medium (MEM), penicillin-streptomycin (PS), trypsin-EDTA, and fetal bovine serum (FBS) were purchased from Gibco (Gibco, NY, USA). MitoTracker Deep Red FM was obtained from Invitrogen (CA, USA), Hoechst 33342 and rhodamine 123 fluorescent probes were obtained from Sigma-Aldrich (MO, USA), and poly-d-lysine was acquired from BBI (Shanghai, China). HEK293 cells were purchased from the Type Culture Collection of the Chinese Academy of Sciences (Shanghai, China). Details of the three positive control medicines and the names of the 18 Chinese HMPs are shown in Table 3. The growth of cultured HEK293 cell was significantly inhibited by 20 *μ*g/mL AA, 5–50 *μ*M CsA, and 10–50 *μ*M CDDP (Bai et al., 2014) [20, 39–41]. In the present study, dose-response effects of the positive control medicines were tested at concentrations of 0.01–100 *μ*M. The 18 Chinese HMPs were serially diluted to 0.01 *μ*M in MEM. NaCl (0.9%) solution and 40% formaldehyde (diluted to 10% in ultrapure water) were purchased from YiFang S&T (Tianjin, China). Serum creatinine (CRE), blood urea nitrogen (BUN), lactate dehydrogenase (LDH), alkaline phosphatase (ALP), malondialdehyde (MDA), thiobarbituric acid, and trace protein detection assay kits were obtained from Nanjing Jiancheng Bioengineering Institute (Nanjing, China).

### 2.2. Cell Culture and Drug Treatment

HEK293 cells were cultured in MEM basal medium with 10% FBS and 1% 100 IU·mL^−1^ PS at 37°C in a 5% CO_2_ atmosphere. Cells were subcultured by trypsinization with 0.25% trypsin-EDTA solution, plating into poly-D-lysine-coated 96-well microplates (Corning, MA, USA) at a density of 1 × 10^4^ per well, and cultured at 37°C in a 5% CO_2_ atmosphere. Only the inner 60 wells were used to avoid edge effects. After 24 h culture, the medium was removed. Serial dilutions of the test or control drugs in 100 *μ*L MEM were added to each well and cultured at 37°C in a 5% CO_2_ atmosphere for 24 h and 48 h.

### 2.3. HCA Image Acquisition and Analysis

The HCA was optimized by determining the number of images to be acquired, the number of imaging channels needed, the objective magnification, and the image pixel size. The number of images acquired depends on the amount of information required and has been previously described by Shariff et al. [42]. In this HCA, CV, NA, and NR were determined with Hoechst 33342. MS and MMP were determined with MitoTracker Deep Red FM and rhodamine 123, respectively. After 24 h or 48 h culture, 50 *μ*L of a fluorophore mixture containing 1.67 *μ*g·mL^−1^ Hoechst 33342 and 0.08 *μ*M MitoTracker Deep Red FM in MEM was added to each well. Cells were incubated for 30 min in the dark. After removing the medium, 100 *μ*L 1.26 *μ*g·mL^–1^ rhodamine 123 was added to each well. The cells were incubated for 30 min in the dark and washed once with warm MEM before performing the image scan. The assay plate was imaged and analyzed with the Operetta HCA system (PerkinElmer, MA, USA) with a 20× objective and a relative humidity of 45% at 25°C. The fluorescent images of nine fields per well in the three fluorescent probe channels were acquired in each confocal scan. The mean values of nine CV, NA, NR, MS, and MMP images were calculated using Columbus (PerkinElmer) and GraphPad Prism (GraphPad Software Inc., CA, USA) software.

### 2.4. Animals and Acute Toxicity Testing

Male 220–250 g Sprague-Dawley rats were purchased from the Laboratory Animal Center of Academy of Military Medical Sciences, housed in metabolism cages under controlled laboratory conditions, and provided with standard rat chow and water ad libitum. They were allowed 1 week of acclimatization at 25 ± 1°C at a 12-hour light/dark cycle. Animal care and experimental procedures followed the China Laboratory Animal Use Regulations and the institutional ethical guidelines. The study was approved by the Animal Care and Use Committee of Tianjin International Joint Academy of Biotechnology and Medicine.

Aristolochic acid (AA), cantharidin (CA), triptolide (TR), sophocarpine (SC), and diosbulbin B (DB) all caused obvious damage to cell nuclei and mitochondria; all five were selected for use in the in vivo experiments. AA and CsA were used as positive controls to verify the accuracy of the HCA results. All samples were dissolved in 0.9% NaCl for injection at 20 mg/kg AA (*n* = 8 per group), 7.5 mg/kg CDDP (*n* = 8 per group), 10 mg/kg DB (*n* = 9 group), 1 mg/kg CA (*n* = 9 group), 0.5 mg/kg TR (*n* = 9 group), and 15 mg/kg SC (*n* = 9 group). These doses were chosen considering previous nephrotoxicity studies and clinical doses used in chemotherapy. A blank control group (*n* = 6 per group) received 0.9% physiological saline. The injected volume was 2 mL and the rats were injected via the tail vein daily for seven consecutive days. The rats were weighed on days 0, 1, 3, 5, and 7 of treatment. They were anesthetized by intraperitoneal injection of 10% chloral hydrate 2 h after the last injection of the treatment drug or saline. Blood samples were collected from the abdominal aorta and kidneys, centrifuged at 1000* g* for 10 min at 4°C, and stored at −80°C until being used in assays. The kidneys were removed, photographed, and imaged by microcomputed tomography (micro-CT; PerkinElmer), an X-ray based instrument designed for preclinical biomedical research for live imaging of small animals. One kidney was frozen in liquid nitrogen and stored at −80°C; the other was fixed in 10% formalin and stored until analysis.

### 2.5. Biochemical Assays

Serum BUN, CRE, LDH, and ALP were assayed with commercial diagnostic kits and an automated biochemical analyzer (Hitachi, Tokyo, Japan). Lipid peroxidation was assayed in 10% w/v homogenates of 0.2 g of renal tissue in cool physiological saline prepared in a Teflon homogenizer followed by centrifugation at 625* g *for 10 min. MDA, an index of fatty acid oxidation, was measured with a thiobarbituric acid reactive substance assay kit as described by Ohkawa et al. [43]. Briefly, MDA in the tissue homogenate reacted with thiobarbituric acid to form a pink complex with maximum absorbance at 532 nm. MDA was reported as nmol/mg protein.

### 2.6. Renal Histopathology

Kidney tissues were fixed in 10% phosphate-buffered formalin, dehydrated in an ascending ethanol series, and embedded in paraffin. The tissue was sectioned at 4 *μ*m, stained with hematoxylin and eosin (H&E), and examined by light microscopy (Nikon 80i, Tokyo, Japan).

### 2.7. Statistical Analysis

Data were expressed as means ± standard error of the mean (SEM). Between-group differences were evaluated by one-way analysis of variance followed by the least significant difference multiple range test. Statistical analysis was performed using the Statistical Package for the Social Sciences software (SPSS, Chicago, IL, USA). *P* values < 0.05 were considered significant.

## 3. Results

### 3.1. Validation of HCA with Positive Control Drugs

**Figure 1:**
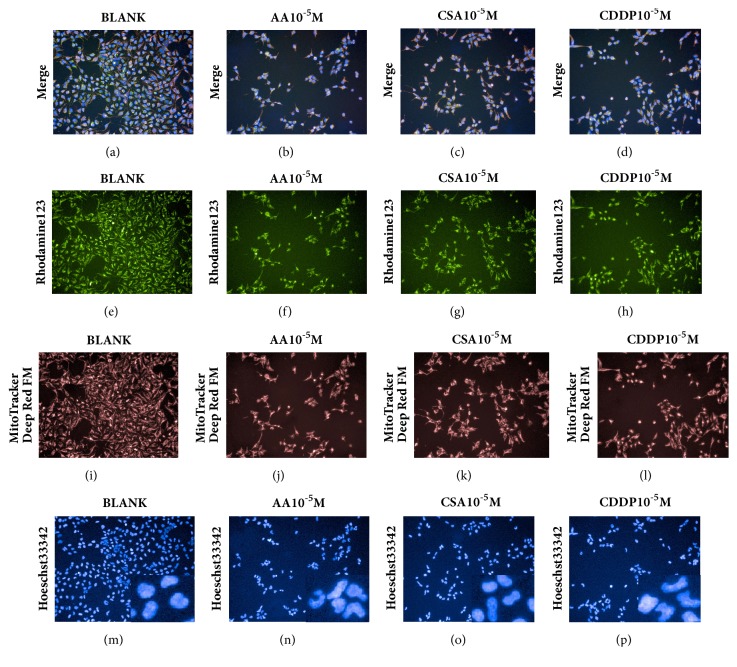
Representative HCA images showing drug-induced nephrotoxicity in Hoechst 33342, MitoTracker Deep Red FM, and rhodamine 123 channels. HEK293 cells treated with blank (a, e, i, m), 10 *μ*M aristolochic acid (AA) (b, f, j, n), 10 *μ*M cyclosporine A (CsA) (c, g, k, o), and 10 *μ*M cisplatin (CDDP) (d, h, l, p) are shown. Decreased intensity of rhodamine 123 indicates decreased mitochondrial membrane potential (MMP) (f, g, h). Increased intensity of MitoTracker Deep Red FM indicates increased mitochondrial mass (MS) (j, k, l). The number of cells stained by Hoechst 33342 was decreased by all three drugs (n, o, p).

**Figure 2:**
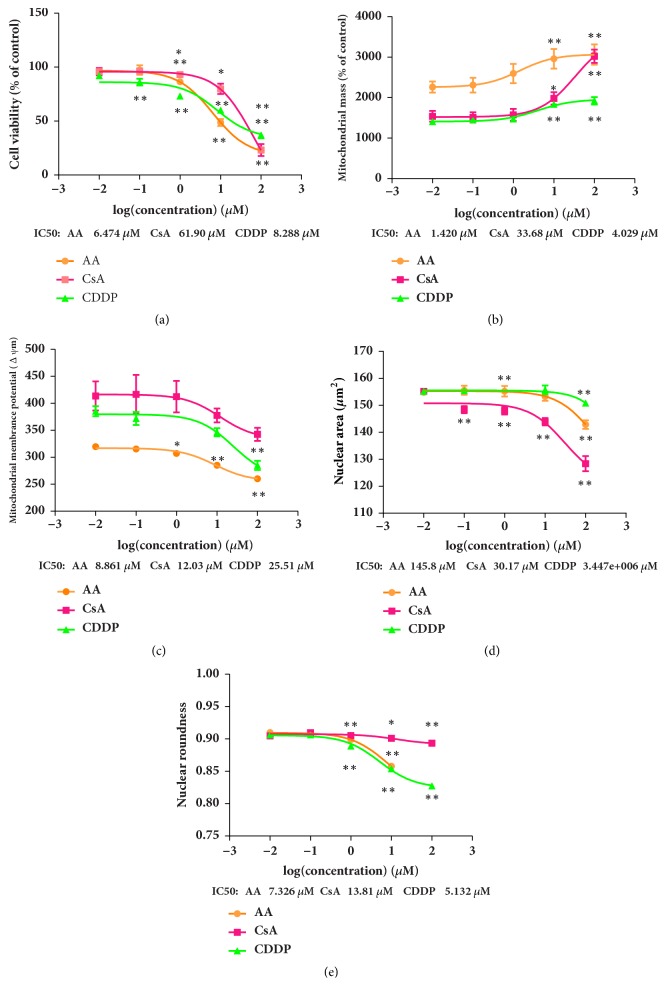
Aristolochic acid (AA), cyclosporine A (CsA), and cisplatin (CDDP) dose-response curves for cell viability (a), mitochondrial mass (b), mitochondrial membrane potential (c), nuclear area (d), and nuclear roundness (e). HEK293 cells were exposed to 0.01, 0.1, 1, 10, and 100 *μ*M of the three drugs. Data are means ± SEM (*n* = 4).

The sensitivity of the multiparametric HCA assay was validated by the three known nephrotoxic compounds. HCA images of control and treated cells are shown in Figure 1. Representative images of the Hoechst 33342, MitoTracker Deep Red FM, and rhodamine 123 channels show the cytotoxic effects of 10***μ***M AA, CsA, and CDDP. Decrease of cell number is shown in Figures 1(b)–1(d). Decreased intensity of rhodamine 123 in Figures 1(f)–1(h) indicates a decrease in MMP, and increased intensity of MitoTracker Deep Red FM in Figures 1(j)–1(l) indicates an increase in MS. Changes in nuclear shape in response to treatment with the positive controls are shown in Figures 1(n)–1(p). The dose response and IC_50_ values of CV, MS, MMP, NA, and NR for AA, CsA, and CDDP are shown in Figure 2.

### 3.2. HCA Nephrotoxicity Assay of Eighteen TCM Compounds.

**Figure 3:**
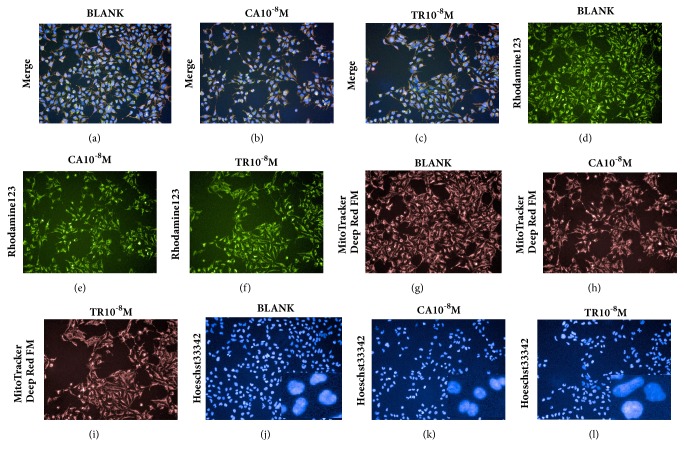
Representative images of the HCA analysis of HEK293 cells for the evaluation of cell number and morphology and stained by three fluorescence probes. Control (a, d, g, j), cyclosporine (CA) 0.01 *μ*M (b, e, h, k), and triptolide (TR) 0.01 *μ*M (c, f, i, l).

**Figure 4:**
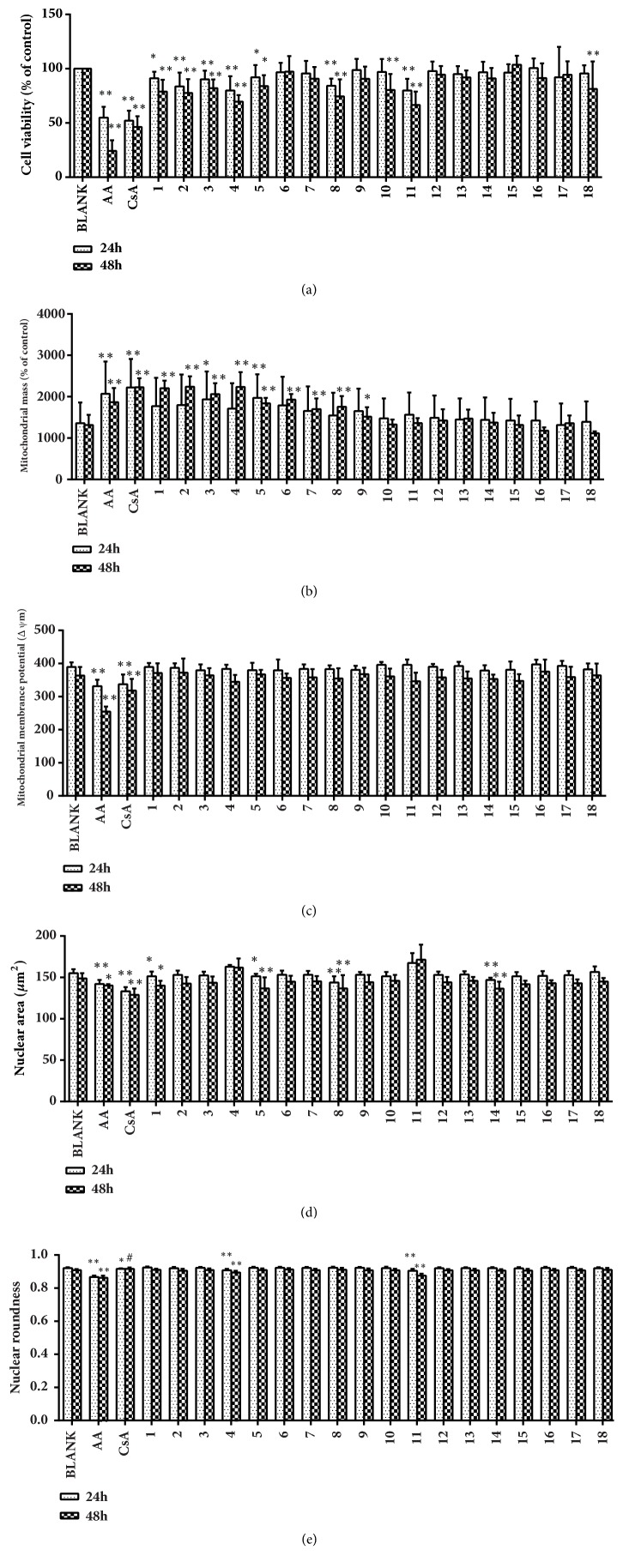
Change of cell viability (CV), nucleus area (NA), nucleus roundness (NR), mitochondrial mass (MS), and mitochondrial membrane potential (MMP) produced in HEK293 cells by 18 compounds after 24 and 48 h culture. Data are means ± SEM (*n* = 3). ^*∗*^*P* < 0.05. ^*∗∗*^*P* < 0.01 compared with controls (BLANK).

**Figure 5:**
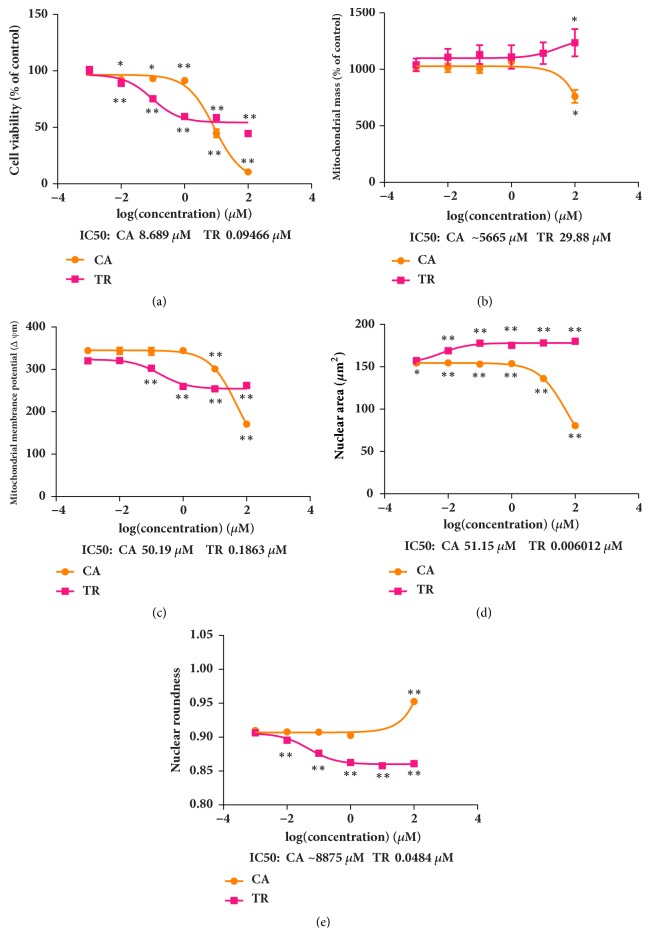
Cantharidin (CA) and triptolide (TR) dose-response curves of cell number (a), mitochondrial mass (b), mitochondrial membrane potential (c), nuclear area (d), and nuclear roundness (e) with exposure to drug concentrations of 0.001, 0.01, 0.1,1, 10, and 100 *μ*M. Data are means ± SEM (*n* = 3).

Eighteen TCM compounds with clear or suspected renal toxicity were tested at a concentration of 0.01 *μ*M. The representative HCA images shown in Figure 3 show the cytotoxic effects on HEK293 cells induced by cantharidin and triptolide. Decrease in the size of the nucleus in response to cantharidin and increase in response to triptolide are apparent in Figures 3(k) and 3(l). The effects of the 18 compounds and comparisons with the blank control are shown in Figure 4. At a concentration of 0.01 *μ*M, cantharidin, diosbulbin B, sophocarpine, triptolide, triptonide, cnidium lactone, and mesaconitine significantly decreased CV. Cantharidin, cnidium lactone, mesaconitine, and periplocin caused nuclear shrinkage, and triptolide and triptonide caused nuclear swelling and decrease of roundness. Cantharidin, diosbulbin B, sophocarpine, triptonide, cnidium lactone, 2,4-acetyl alisma alcohol A, 2,3-acetyl alisma alcohol B, mesaconitine, and hypaconitine caused an increase in MS. The effects of these compounds were generally greater at 48 h than at 24 h. Tripterine and rhein had no effect at 24 h but significantly decreased CV after 48 h. As cantharidin and triptonide had the strongest influence on the cell nucleus and mitochondria, they were selected for an evaluation of the concentration-response relationship over a range of 0.001–100 *μ*M. The IC_50_ concentrations for the detection indexes are shown in Figure 5.

### 3.3. Renal Effects of Acute Toxicity in Rats

**Figure 6:**
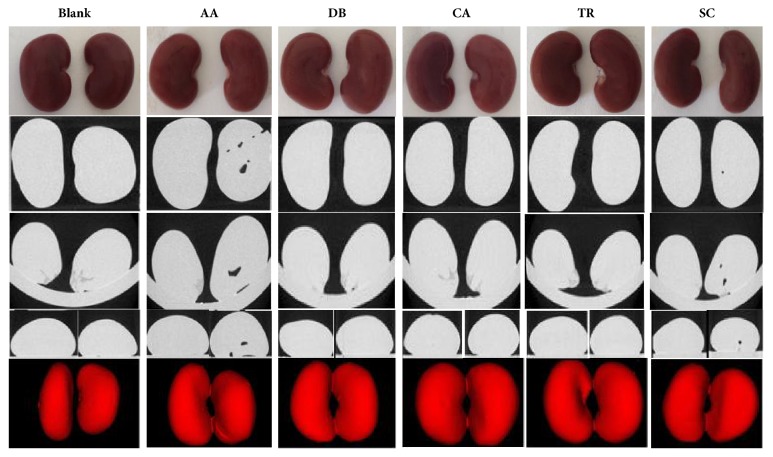
Photographs and micro-computed tomography scans showing the effects of nephrotoxic agents including aristolochic acid (AA), diosbulbin B (DB), cantharidin (CA), triptolide (TR), and sophocarpine (SC) on kidney shape, symmetry, and structure.

The HCA results were confirmed by evaluating acute toxicity in rats. Of the 58 animals used to test acute toxicity, six died, three after day 4 of CDDP injection and three after day 5 of TR injection (Table 4). The kidneys were photographed and imaged with micro-CT scanning. Micro-CT provided a view of the three-dimensional architecture of basic functional units of tissue including intact nephrons. The size, shape, and symmetry of the kidneys in the control group appeared to be normal (Figure 6). Kidneys from the animals of the five experimental groups showed signs of acute toxicity, including asymmetry of size or shape. Kidneys from the rats treated with AA and SC group had spaces within the kidney, indicating irreparable damage.

### 3.4. Biochemistry

**Figure 7:**
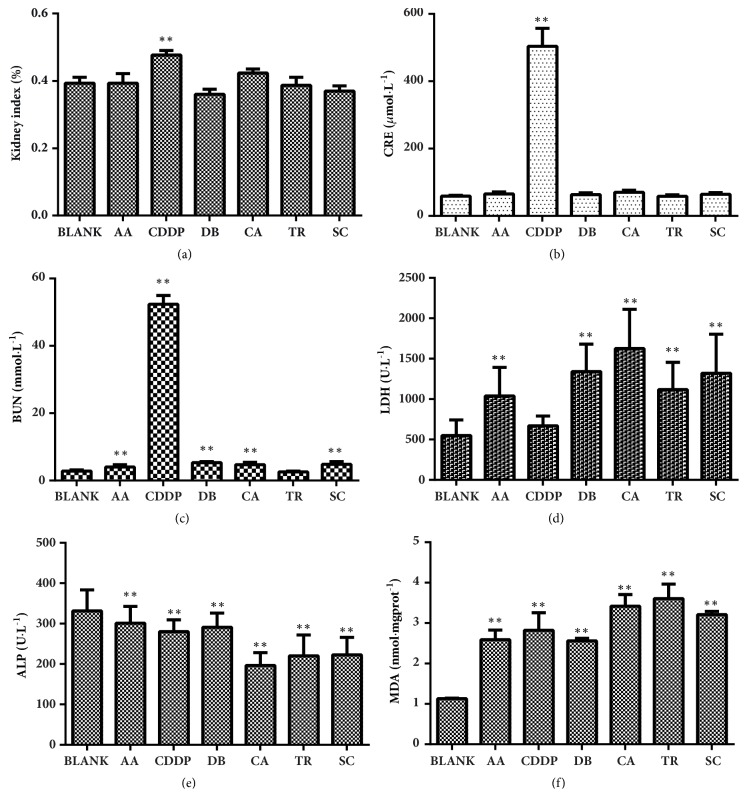
The effect of aristolochic acid (AA), cisplatin (CDDP), diosbulbin B (DB), cantharidin (CA), triptolide (TR), and sophocarpine (SC) on the kidney index (a), CRE (b), BUN (c), LDH (d), and ALP (e) in serum and MDA (f) in renal cortical homogenates. Data are means ± SEM (*n* = 4). ^*∗*^*P* < 0.05; ^*∗∗*^*P *< 0.01 compared with controls (BLANK).

The kidney/body weight ratio (kidney index) and CRE levels of rats injected with AA, DB, CA, TR, or SC and blank control animals were not significantly different, but the kidney index and CRE were both significantly higher in rats given CDDP than in controls (Figure 7). Except for TR, BUN was significantly increased, and except for CDDP, LDH was significantly increased compared with controls. Serum ALP concentration was significantly decreased and MDA concentration in kidney homogenates was significantly increased in all the experimental groups compared with controls. Acute renal dysfunction and kidney injury occurred in rats treated with the selected Chinese compounds.

### 3.5. Renal Histopathology

**Figure 8:**
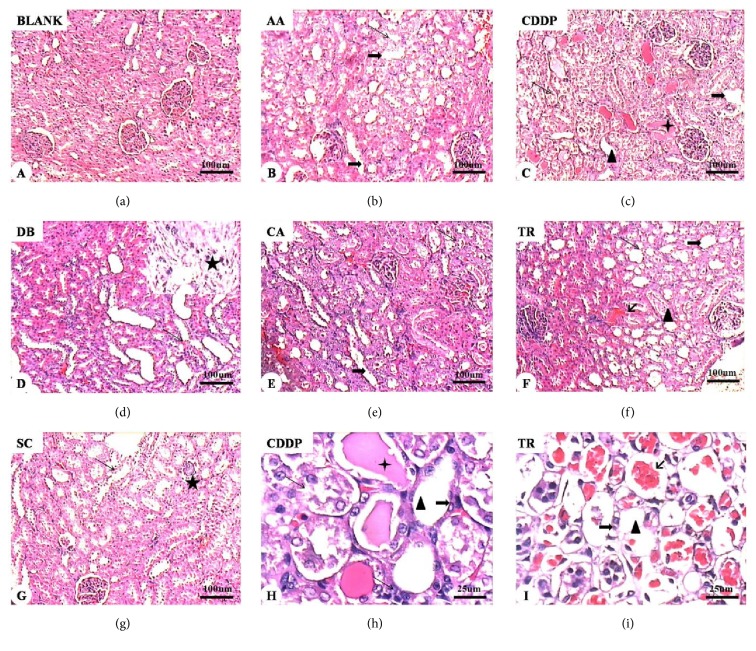
Histology of renal tissue. Normal histological characteristics of glomeruli and tubules of the control group (a). The major histological changes in the experimental groups include increased acidophilia, cystic dilation of renal tubules, loss of the proximal brush border, vacuole degeneration, pyknotic or swollen nuclei, and diffuse particles in epithelial cells (long arrows in (e)–(i)). Hyaline (triangles in (c), (f), (h), and (i)), hematic (crosses in (c) and (h)), and erythrocyte (short arrows in (f) and (i)) casts are present inside renal tubules. Naked renal tubular basement membranes can be seen (thick arrows in (b), (c), (e), (f), (h), and (i)). Asterisks in (d) (enlarged view) and (g) show renal tubular epithelial cell necrosis, sloughing, and small calcified bodies. H&E stain. Magnification: ×10 (a–g) and ×40 ((h) and (i)).

**Figure 9:**
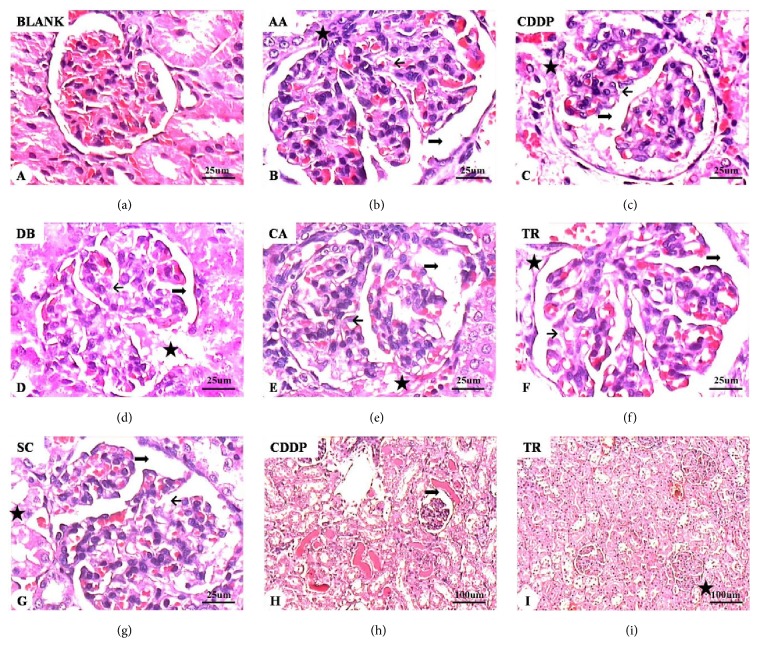
Representative micrographs of renal glomerular histology. Control group: normal glomerular morphology (a). Experimental groups: aristolochic acid (AA) (b), cisplatin (CDDP) (c, h), diosbulbin B (DB) (d), cantharidin (CA) (e), triptolide (TR) (f, i), and sophocarpine (SC) (g). Normal glomerular morphology from control animals (a) and altered glomerular morphology in drug-treated rats (b–i). Enlarged glomeruli, increased cell number, and lobulated glomeruli (b–h). Mesangial cell proliferation, increased intercellular matrix, and dilatation of Bowman's space (thick arrows in (b)–(h)). The capillary loops are open or collapsed closed (short arrows in (b)-(g)). Red dye foam sample exudate can be seen in the renal capsule. Epithelial hyperplasia and glomerular adhesions with the renal capsule (asterisks in (b)–(g) and (i)). H&E stain. Magnification: ×10 ((h) and (i)) and ×40 (a–g).

Morphological and histological changes associated with drug treatment were evaluated in H&E-stained kidney tissue (Figures 8 and 9). Renal tubules and glomeruli appeared to be normal in control rats. Progressive, degenerative lesions with acute tubular necrosis and multifocal necrosis were seen in the experimental rats treated with the six compounds (Figure 8). Histological evaluation revealed atrophy of tubular epithelial cells, fatty degeneration and tubular dilatation, hyperemia calcification, disruption of tubule basement membranes, protein casts, glomerular enlargement, and lobulated glomerular tufts. Granular degeneration and coagulation were apparent in the renal tubule epithelia, most notably after treatment with AA, CDDP, and TR. The most severe damage of renal tubular epithelium was seen in CDDP-treated rats. The results indicated that CDDP caused the most serious renal damage followed by TR and AA.

## 4. Discussion and Conclusion

Chinese traditional medicine has been practiced for 2000 years, but investigation of the associated side effects has just started. A characteristic pathological feature of drug-induced nephrotoxicity is the death of renal tubule cells, which involves both necrosis and apoptosis in various tubular segments. The intrinsic, mitochondria-mediated pathway of apoptosis has also been implicated in drug-induced renal injury [20]. HCA has a high flux capacity and it can not only clarify the biological activity of the agents being tested but also confirm the interaction between drugs and their targets. Used to screen for potential toxicity, cell-based HCA offers benefits of rapid, high flux detection which can predict drug-induced toxicity and also reduce the dependence on toxicity studies in animal models. It can also be applied in drug discovery projects to optimize the safety of compound series and provide a risk assessment of the candidate compounds [44]. Multiparameter HCA assays have been used to measure genotoxicity, cardiotoxicity, hepatotoxicity, drug-induced phospholipidosis, and developmental neurotoxicity. The sensitivity of HCA systems has been demonstrated, but HCA-based nephrotoxicity screening has been challenging to evaluate drug combinations, especially for provision of organ-specific toxicity profiles and an understanding of the underlying mechanisms, because of their complex chemical properties and safety evaluations of TCM preparations. Compared with conventional assays, the increased sensitivity of HCA provides clear images of cells and quantifies multiple cellular biomarkers to evaluate the toxic effects of compounds and analyze the pathogenesis of toxicity [45]. This study used a multiparametric assay of markers within a single cell image, or multi-index imaging, to detect renal toxicity. Median lethal doses in line with previous reports and screening data of 18 species of single molecules with known renal toxicity validated the accuracy of the HCA method. The speed and accuracy of monomer screening, comprehensive evaluation index, precise imaging of nuclear morphology, and analysis of nuclear and mitochondrial function allowed a preliminary analysis of the renal toxicity of 18 TCM components. It can be successfully applied to the evaluation of toxicity and the selection of other TCM drugs.

AKI has high morbidity and cost burdens, and a large proportion of patients progress to chronic renal failure requiring dialysis. One of the etiologies of AKI is nephrotoxicity drugs, including cisplatin, cyclosporine A, AA, gentamicin, HgCl_2_, and glycerol, and all of them have cytotoxic effects on renal tubular cells. Mouse models are widely employed in preclinical studies; few urinary biomarker studies have been performed in mice because of limited urine production and lack of sensitive assays. Serum creatinine and BUN are the markers of choice in preclinical animal studies of kidney injury together with histopathology. However, creatinine is not a reliable indicator of acute changes in kidney function, as its concentration might not change until 25%–50% of kidney function has already been lost. It is also not reflective of glomerular filtration rate owing to a number of renal and nonrenal influences. In this study, BUN and serum CRE and LDH and ALP activity were assayed. Compared with the controls, the tested compounds significantly changed almost all the indexes. CDDP caused significant increases in both the kidney index and CRE but not the other markers, indicating that it produced more serious kidney damage than the other compounds did.

Under normal conditions, oxygen free radical production and lipid peroxidation reactions are active in maintaining metabolic homeostasis and in regulating immune and other physiological responses. If coordination and dynamic balance are lost, a series of metabolic and immune function disorders result in damage of kidney tubule epithelium and changes in permeability resulting in cell, tissue, and organ damage, as well as fibrosis. Interventions that can prevent or decrease production of free radicals might reduce oxidative damage and necrosis induced by nephrotoxicity drugs. In addition, peroxidation of membrane phospholipids increases membrane fluidity and permeability and results in swelling of renal tubule cells and degeneration of cell organelles, such as the nucleus and mitochondria [46]. Almost all of the drugs tested induced a significant elevation in MDA, a secondary product of lipid peroxidation.

In this study, the main findings of the kidney biopsies were acute tubular necrosis and glomerular abnormalities. The experimental animals had histological alterations compatible with acute tubular necrosis, including pyknotic nuclei, epithelial cell necrosis, loss of basement membranes, and hyaline and hematic casts within tubules. Increases in lobulated glomeruli and cell number were seen in all experimental groups. Mesangial cell proliferation, increased intercellular matrix, expansion of Bowman's space, and dilated or collapsed capillary loops were also observed. Hyperplasia of the kidney capsule epithelium and adhesions of the glomerular capillaries with the renal capsule were also seen.

The present study used a novel HCA system that was predictive of drug-induced kidney injury. HCA of the effects on HEK293 cells and the acute effects in rats confirmed the nephrotoxicity of cantharidin, triptolide, diosbulbin B, and sophocarpine present in traditional Chinese medical products. HCA was an effective toxicity screening test and can contribute to the clinical safety of the drugs used in TCM.

## Figures and Tables

**Table 1:** Source of toxic components.

**Component name**	**The source of plants or animals**	**Chinese name**
Cantharidin	*Mylabris*	Banmao
Diosbulbin B	*Dioscorea bulbifera* rhizoma	Huangyaozi
Sophocarpine	*Sophorae flavescentis* radix	Kushen
Triptonide	*Tripterygium wilfordii* radix et rhizoma	Leigongteng
Cnidium lactone	*Fructus cnidii*	Shechuangzi
2,4-acetyl alisma alcohol A	Rhizoma Alismatis	Zexie
2,3-acetyl alisma alcohol B	Rhizoma Alismatis	Zexie
Mesaconitine	Radix Aconiti, Radix Aconiti Kusnezoffii, Radix Aconiti Lateralis Praeparata	Chuanwu Caowu Fuzi
Hypaconitine	Radix Aconiti, Radix Aconiti Kusnezoffii, Radix Aconiti Lateralis Praeparata	Chuanwu Caowu Fuzi
Tripterine	*Tripterygium wilfordii* radix et rhizoma	Leigongteng
Triptolide	*Tripterygium wilfordii* radix et rhizoma	Leigongteng
Evodiamine	Fructus Evodiae	Wuzhuyu
Rutaecarpine	Fructus Evodiae	Wuzhuyu
Periplocin	Cortex periplocae	Xiangjiapi
Periplocymarin	Cortex periplocae	Xiangjiapi
Periplogenin	Cortex periplocae	Xiangjiapi
Emodin	Polygoni Cuspidati rhizoma et radix, Rhei radix et rhizoma	Huzhang Dahuang
Rhein	Rhei radix et rhizoma	Dahuang

**Table 2:** The toxicity classification of TCMs involved this study in the Pharmacopoeia.

**Classification**	**Traditional Chinese medicine**
Strong toxicity	Radix Aconiti Kusnezoffii, Caowu
	*Mylabris*, Banmao
	Radix Aconiti, Chuanwu

Toxical	Radix Aconiti Lateralis Praeparata, Fuzi
	Cortex periplocae, Xiangjiapi
	*Tripterygium wilfordii* radix et rhizoma, Leigongteng

Mild toxicity	Fructus Evodiae, Wuzhuyu
	*Fructus cnidii*, Shechuangzi
	*Dioscorea bulbifera* rhizoma, Huangyaozi

**Table 3:** The medicines that were evaluated.

**Number**	**Component name**	**Purchased from**
Positive medicines	Aristolochic acid	Zhongxin Innova®, China
	Cyclosporine A	Gene Operation, USA
	Cisplatin	Sigma, USA

1	Cantharidin	Zhongxin Innova, China
2	Diosbulbin B	Zhongxin Innova, China
3	Sophocarpine	Zhongxin Innova, China
4	Triptonide	Zhongxin Innova, China
5	Cnidium lactone	Zhongxin Innova, China
6	2,4-acetyl alisma alcohol A	Zhongxin Innova, China
7	2,3-acetyl alisma alcohol B	Zhongxin Innova, China
8	Mesaconitine	Zhongxin Innova, China
9	Hypaconitine	Zhongxin Innova, China
10	Tripterine	Zhongxin Innova, China
11	Triptolide	Zhongxin Innova, China
12	Evodiamine	Zhongxin Innova, China
13	Rutaecarpine	Zhongxin Innova, China
14	Periplocin	Zhongxin Innova, China
15	Periplocymarin	Zhongxin Innova, China
16	Periplogenin	Zhongxin Innova, China
17	Emodin	Zhongxin Innova, China
18	Rhein	Zhongxin Innova, China

**Table 4:** Pathological changes associated with acute toxicity.

**Group**	**Death**/**total animals**	**Abnormalities**
Control	0/6	Normal
Aristolochic acid (AA)	0/8	Rats have dark yellow hair
Cisplatin (CDDP)	3/8	Rats have dark yellow hair, dispirited
Diosbulbin B (DB)	0/9	Rats have dark yellow hair
Cantharidin (CA)	0/9	Rats have dark yellow hair
Triptolide (TR)	3/9	Rats have dark yellow hair, dispirited, huddle up
Sophocarpine (SC)	0/9	Rats have dark yellow hair

## Data Availability

The data used to support the findings of this study are available from the corresponding author upon request.
